# Treatment of residual and phantom limb pain using percutaneous ablation: a systematic review and meta-analysis

**DOI:** 10.3389/fresc.2026.1802455

**Published:** 2026-04-13

**Authors:** Kevin R. Murray, Griffin Pauli, Michael Catapano

**Affiliations:** 1 Division of Physical Medicine and Rehabilitation, Department of Medicine, University of Toronto, Toronto, ON, Canada; 2 Division of Neurology, Department of Medicine, University of Toronto, Toronto, ON, Canada; 3 Division of Orthopedic Surgery, Department of Surgery, University of Toronto, Toronto, ON, Canada

**Keywords:** amputation, chronic pain, cryoablation, phantom limb pain, radiofrequency ablation

## Abstract

**Introduction:**

Limb loss is estimated to affect 176 million people globally. Up to 80% of patients experience significant residual limb pain (RLP) and phantom limb pain (PLP) following amputation, and generally respond poorly to conventional therapies. Percutaneous ablation has emerged as a promising intervention for both RLP and PLP pain. We sought to systematically review and evaluate the efficacy and adverse outcomes of thermal radiofrequency ablation (RFA), pulsed RFA, and cryoablation in the treatment of post-amputation pain.

**Methods:**

Medline, Embase, PubMed, and Scopus databases were systematically search on August 19, 2024 for all English language articles related to post-amputation pain treated with an ablation technique. Articles were assessed for risk of bias using the Joanna Briggs Institute tool for case reports and case series. Outcomes included response to standardized pain scales (either numeric rating scale or visual analog scale), functional outcomes, and adverse events.

**Results:**

Out of 1233 articles, 20 were included for analysis. This included 1 randomized control trial (*n* = 144 patients), 9 case series (*n* = 74), and 10 case reports (*n* = 10). Comparing across 3 studies (*n* = 32 patients), thermal radiofrequency ablation (RFA) improved RLP compared to baseline at 2-weeks [weighted mean difference from baseline: 7.0 (95% CI: 6.8–7.3; *p* < 0.0001)] and 6-month [6.7 (6.7–6.8; *p* < 0.0001)]. For pulsed RFA we compared across 4 studies (*n* = 6 patients) to show improvement in RLP from baseline to 1-month [7.8 (7.1–8.5; *p* < 0.0001)] and 6-months [6.4 (4.7–8.1; *p* < 0.0001)], as well as across 7 studies (*n* = 10 patients) to show improvement in PLP from baseline at 1-month [5.9 (4.0–7.7; *p* < 0.0001)] and 6-months [5.6 (3.7–7.5); *p* < 0.0001]). In 3 studies (*n* = 27 patients) cryoablation decreased PLP compared to baseline at 6-months [3.9 (3.7–4.1; *p* < 0.0001), but a randomized control trial (*n* = 144 patients] did not demonstrate significant benefit at 4-months. There was one report of a fall secondary to temporary motor block with no other significant complications reported across all studies.

**Conclusions:**

Both thermal and pulsed RFA appear to be safe and effective treatments for post-amputation pain. Further research is required to better assess their effectiveness and safety profile in larger and more well controlled studies.

## Introduction

Limb loss is estimated to affect 176 million people globally, accounting for 5.5 million years lived with disability (1). Given the increasing prevalence of cardiometabolic disease, it is expected that the rate of amputations in the United States will double by 2050 and over 1% of Americans will be living with limb loss (2). While the biomechanical impact of limb loss directly affects patient function, post-amputation pain is associated with decreased quality of life and limits functional independence through decrease prosthetic tolerance (3). Following amputation, over half of patients experience chronic post-amputation pain, which includes both residual limb pain (RLP) and phantom limb pain (PLP) (4). RLP includes nociceptive pain at the site of amputation secondary to local tissue damage, heterotopic ossification, scar formation, and referred pain from altered biomechanics, as well as local neuropathic pain from proximal neuroma formation. Alternatively, PLP is a predominantly neuropathic phenomenon, in which pain is experienced in the distribution of the amputated limb (5). Despite its original description by Ambrose Pare in 1552 (6), the pathophysiology of PLP remains and active area of study with contribution from both peripheral (e.g., neuroma formation) and central (e.g., central sensitization and cortical reorganization) factors suspected (5).

No clear evidence-based treatment guidelines exist for the management of post-amputation pain. A 2016 Cochrane review assessing pharmacological interventions for PLP identified an unclear impact of common neuropathic pain medications on PLP secondary to a lack of large and well controlled studies (7). Similarly, expert consensus has highlighted mixed evidence and only modest improvements in PLP following pharmacotherapy treatment (8), leaving patients with limited options for pain management.

Two modern surgical techniques, targeted muscle reinnervation (TMR) and regenerative peripheral nerve interface (RPNI), have emerged as promising interventions for the treatment of post-amputation pain. In TMR, transected mixed or motor nerves are grafted to healthy denervated muscle allowing the nerve to grow and innervate a new territory (9). Alternatively, RPNI involves securing a denervated and dysvascular muscle graft around a transected sensory nerve that is not a candidate for TMR (9). While these techniques can be used prophylactically at the time of amputation, surgical re-intervention for patients with pre-existing amputations, particularly those with a vascular etiology, caries a significant risk of complications and poor healing (9). As a result, percutaneous treatment options may represent a safer alternative to surgery.

A recent narrative review of post-amputation pain in lower extremity amputees was supportive of several percutaneous techniques, including stimulation, immunosuppression, botulinum toxin, alcohol neurolysis and ablation, however lacked a systematic and comprehensive review on the literature (10). The purpose of this study was to comprehensively assess the effectiveness and safety of percutaneous ablation for the treatment of post-amputation pain in patients with upper or lower extremity limb loss.

## Materials and methods

### Search strategy

This study was designed in accordance with the Preferred Reporting Items for Systematic Review and Meta-analysis guidelines and reviewed by a medical sciences librarian (11). On August 19, 2024, Medline, Embase, PubMed, and Scopus databases were searched for studies related to ablation procedures for post-amputation pain. Search terms included those related to “amputation”, “pain”, and “ablation”.

### Assessment of eligibility

We included randomized control trials, case series, and case reports that reported the use of a percutaneous ablation technique [e.g., thermal radio frequency ablation (RFA), pulsed RFA, or cryoablation] in patients with post-amputation pain. Conference abstracts and brief reports were excluded from statistical analysis as they did not provide sufficient data. Studies that were classified as reviews, editorials, or animal models were excluded.

### Risk of bias assessment

Risk of bias assessment was completed using the Joanna Briggs Institute risk of bias tool for case reports (12) and case series as appropriate (13). Risk of bias for the single randomized control trial was assessed using the Cochrane Risk of Bias 2 tool for crossover trials (14). Assessments were completed in duplicate. Discordance between reviewers was resolved by consensus or independent assessment by an additional reviewer when required.

### Screening and data extraction

Title, abstract screening and full text review was completed in duplicate. For title and abstract screening, discordance between reviewers resulted in article inclusion in full text review to prevent premature exclusion. During full-text review, disagreements were resolved via consensus. Reference lists of included studies were screened for additional relevant articles. Data extraction of included studies was completed in singlet.

### Statistical analysis

Unweighted kappa was calculated to assess agreement of study eligibility at the title, abstract, and full-text screening stages between reviewers. Kappa values are categorized as substantial agreement (greater than 0.59), moderate agreement (between 0.59 and 0.21), or slight agreement (less than 0.21) (15). For individual case reports, visual analog or numeric rating scale scores for PLP and/or RLP were extracted. Where a baseline pain score was reported, but only percent change was reported at follow up, raw pain scale score was calculated for each time point. If a range of pain scores was provided, the mean value was used for analysis. For case series, visual analog or numeric rating pain scale score mean and variability [i.e., standard deviation, range, or 95% confidence intervals (CI)] was extracted.

All timepoints that were reported in 3 or more studies were utilized for our meta-analysis. For case series and reports assessing thermal RFA, baseline, 2-weeks and 6-month time points were used for meta-analysis. For studies assessing pulsed RFA or cryoablation, baseline, 1–3-months, and 6-month timepoints were used for meta-analysis. For the two studies (16, 17) that reported response to cryoablation at both 1-month and 3-months, a mean value of the two time points was calculated for each study and used for the meta-analysis. Weighted mean differences in pain scores were calculated for the above time points for both RLP and PLP and reported with 95% CI. Comparisons between time points were made using Student's *t-*tests in Excel. Results from the single randomized control trial were not included in the pooled analysis and reported separately.

## Results

### Search results

**Figure 1:**
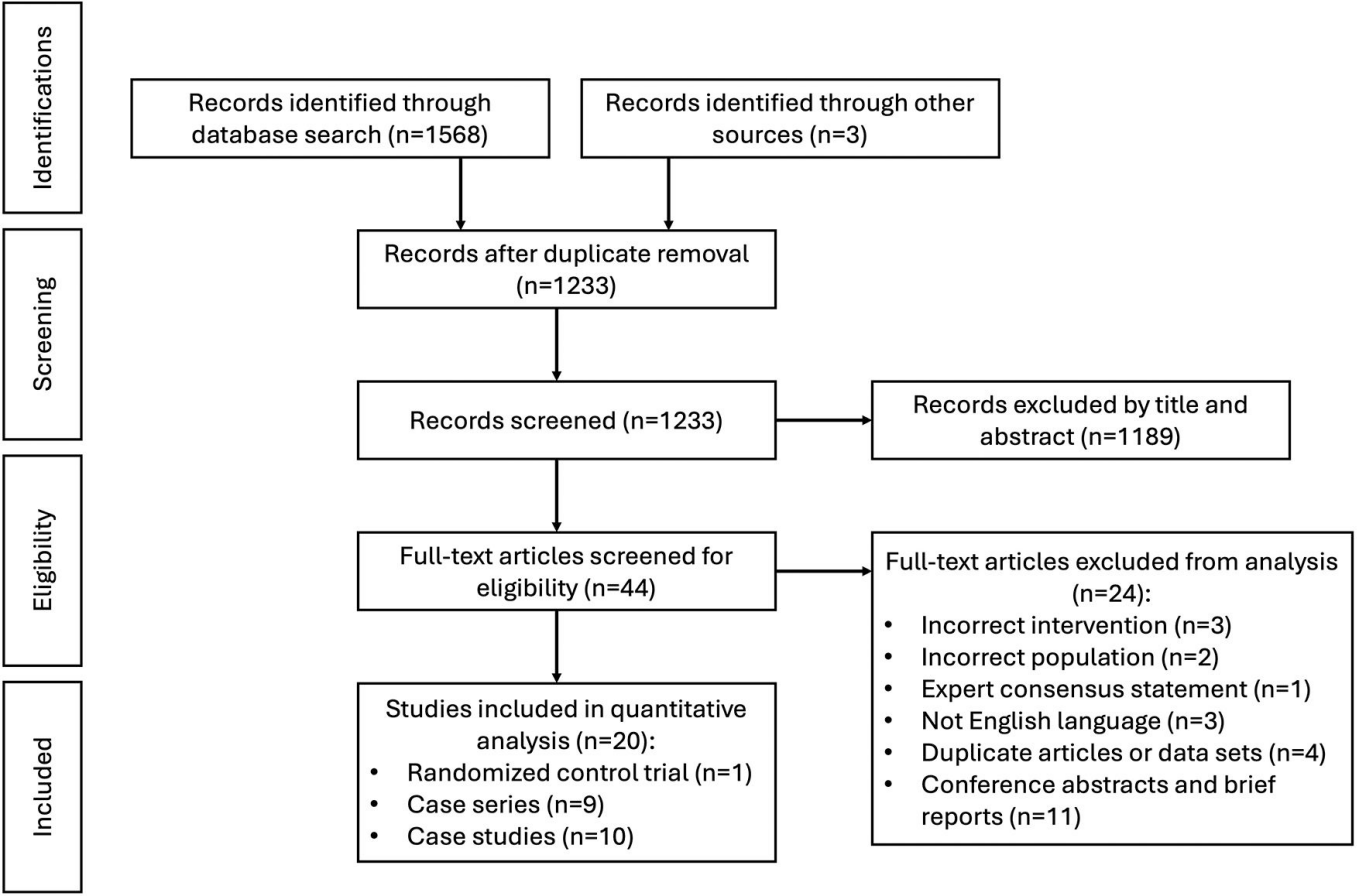
Systematic review data flow diagram.

The search returned 1230 results after duplicate removal. During title screening 1154 articles were excluded (kappa = 0.84), and an additional 35 articles were excluded during abstract screening (kappa = 0.84). During full text review, 24 articles were excluded (kappa = 1.0). Three additional articles were included from reference list screening, resulting in the inclusion of a total of 20 studies. These included 1 randomized control trial (*n* = 144 patients), 9 case series (*n* = 74), and 10 individual case reports (*n* = 10; Figure 1; Table 1).

**Table 1:** Summary of study characteristics.

Study	Study Size (*n*)	Amputation	Amputation Etiology	Anatomical Target	Guidance Technique	Technique	Ablation Duration (min)	Ablation Cycles	Ablation Temperature (°C)
Pu et al. (20)	18	Shoulder (*n* = 1)	Trauma (*n* = 15)	Brachial plexus neuroma (*n* = 1)	Ultrasound and stimulation	Thermal	2	1	80
		Transhumeral (*n* = 1)							
			PVD (2)						
				Median neuroma (*n* = 3)					
			Thrombo-embolic (*n*−1)						
		Transradial (*n* = 3)							
				Ulnar neuroma (*n* = 1)					
		Transfemoral (*n* = 8)							
				Radial neuroma (*n* = 1)					
		Transtibial (*n* = 5)							
				Digital neuroma (*n* = 1)					
				Sciatic neuroma (*n* = 8)					
				Tibial neuroma (*n* = 5)					
				Fibular neuroma (*n* = 3)					
Sperry et al. (21)	1	Transtibial	Neoplasm	Sciatic neuroma	Ultrasound and fluoroscopy	Thermal	2.5	1	60
Guo et al. (19)	9	Transradial (*n* = 1)	Trauma (*n* = 4)	Ulnar neuroma (*n* = 1)	Ultrasound	Thermal	9	1	90
		Hip (*n* = 1)							
			PVD (*n* = 2)						
		Transfemoral (*n* = 2)							
				Sciatic neuroma (*n* = 3)					
			Neoplasm (*n* = 2)						
		Transtibial (*n* = 5)							
			Infection (*n* = 1)						
				Tibial neuroma (*n* = 3)					
				Common fibular neuroma (*n* = 1)					
				Superficial fibular neuroma (*n* = 1)					
Zhang et al. (18)	5	Transradial (*n* = 1)	NR	Peripheral neuroma	Ultrasound and stimulation	Thermal	1	2	80
		Transfemoral (*n* = 3)							
		Transtibial (*n* = 1)							
Fiala et al. (26)	1	Toe	Thromobo-embolic	Tibial nerve		Cryoablation	8, 3	2	NR
von Falck et al. (22)	7	Transfemoral (*n* = 2)	NR	Peripheral neuroma	Ultrasound	Cryoablation	6	2	NR
		Transtibial (*n* = 5)							
Prologo et al. (23)	21	Shoulder (*n* = 2)	Trauma (*n* = 9)	Peripheral neuroma (*n* = 13)	Ultrasound and computed tomography	Cryoablation	10	2	−40
		Transhumeral (*n* = 1)							
			PVD (*n* = 7)						
		Transradial (*n* = 1)		NR (*n* = 8)					
			Neoplasm (*n* = 3)						
			Thrombo-embolic (*n* = 2)						
		Transfemoral (*n* = 10)							
		Transtibial (*n* = 7)							
Ramsook et al. (25)	1	Hip	Trauma	Peripheral neuroma	Ultrasound	Cryoablation	NR	NR	NR
Moesker et al. (24)	5	Upper extremity (*n* = 2)	Trauma (*n* = 2)	NR	Stimulation	Cryoablation	3	2	−70
		Lower extremity (*n* = 3)	PVD (*n* = 3)						
Brzeziński et al. (31)	1	Transtibial	PVD	Femoral and sciatic nerves	Ultrasound and stimulation	Pulsed	2.5	3	42
Li et al. (32)	1	Upper extremity	NR	C6-C8 nerve roots	Stimulation	Pulsed	0.5	2	NR
Zheng et al. (16)	1	Transhumeral	Trauma	Brachial plexus neuroma	Ultrasound and stimulation	Pulsed	4	3	42
Zeng et al. (17)	1	Transfemoral	Trauma	Femoral neuroma and sciatic nerve	Ultrasound and stimulation	Pulsed	NR	1	NR
Kim et al. (33)	1	Transtibial	NR	Sciatic neuroma	Ultrasound	Pulsed	3	3	42
Imani et al. (28)	2	Hemipelvectomy (*n* = 1)	Trauma (*n* = 1)	L4 and L5 DRG	Fluoroscopy	Pulsed	2	2	42
		Transfemoral (*n* = 1)	Neoplasm (*n* = 1)						
Restrepo-Garces et al. (34)	1	Transtibial	Trauma	Sciatic neuroma	Ultrasound and stimulation	Pulsed	2	2	42
West et al. (29)	4	Transhumeral (*n* = 1)	Trauma (*n* = 4)	Femoral neuroma (*n* = 1)	Stimulation	Pulsed	2	2	42
		Transfemoral (*n* = 3)							
				Tibial neuroma (*n* = 1)					
				Fibular neuroma (*n* = 1)					
				NR (*n* = 1)					
Ramanavarapu et al. (30)	2	Transtibial (*n* = 2)	Trauma (*n* = 1)	L4 and L5 DRG	Fluoroscopy and stimulation	Pulsed	2	1	42
			Infection (*n* = 1)						
Wilkes et al. (35)	1	Transfemoral	PVD	Sciatic nerve	Stimulation	Pulsed	2	2	42

PVD, peripheral vascular disease; DRG, dorsal root ganglion, and NR, not reported.

### Risk of bias

The risk of bias assessments for case reports and case series are summarized in Supplementary Tables S1, S2, respectively. Among case reports, common areas for bias included reporting patient demographics and history (4 studies), diagnostics assessment (2 studies), and complications (7 studies). In case series, common domains for bias included participant inclusion (7 studies), patient demographics and history (2 studies) diagnostic assessment (2 studies), and statistical analysis (1 study). Risk of bias assessment for the single randomized control trial is summarized in Supplementary Table S3, and indicates an overall moderate risk related to a lack of reporting on the statistical analysis.

### Thermal RFA

A total of 3 case series (18–20) and 1 case report (21) utilized thermal RFA (Table 2). Across all studies, trauma was the most common etiology of amputation (*n* = 19), followed by peripheral vascular disease (PVD; *n* = 4), neoplastic (*n* = 3), thromboembolism (*n* = 1) and infection (*n* = 1). Anatomical targets for ablation included neuromas of the brachial plexus (*n* = 1) and median (*n* = 3), ulnar (*n* = 2), radial (*n* = 1), digital (*n* = 1), sciatic (*n* = 12), tibial (*n* = 8), and fibular nerves (*n* = 5).

**Table 2:** Response of phantom and residual limb pain and functional outcomes to thermal radiofrequency ablation.

Study	Study Size (n)	PLP Severity (mean score/10)			RLP Severity (mean score/10)			Additional outcome(s)
		BL	2 w	6 m	BL	2 w	6 m	
Pu et al. (20)	18	9.3	2.2	2.9	8.6	1.2	1.9	Frequency of PLP (events/week): BL 8.1; 2 w 1.5; 6 m 2.5
Sperry et al. (21)	1	—	0	—	—	—	—	None
Guo et al. (19)	9	—	—	—	9.6	3.5	—	Improved prosthetic use (6/9), decreased analgesic needs in patients that responded to intervention (3/6), and complete cessation of PLP (3/6)
Zhang et al. (18)	5	^—^	^—^	—	8.6	1.2	1.75	Improved frequency of intermittent pain at 2 w (5/5) and decreased severity of PLP at 2 w (3/3)

PLP, phantom limb pain; RLP, residual limb pain; BL, baseline; m, month; and w, weeks.

Across 3 studies (*n* = 32 patients) (18–20) RLP was improved from baseline at 2-weeks [weighted mean difference 7.0 (95% CI 6.8–7.3); *p* < 0.0001] and 6-months [6.7 (95% CI 6.7–6.8); *p* < 0.0001].

### Cryoablation

There were 3 case series (22–24), 2 case reports (25, 26), and 1 randomized control trial (27) that assessed cryoablation (Table 3). Most patients had an amputation secondary to trauma (*n* = 12) or PVD (*n* = 10), with other etiologies including neoplasm (*n* = 3), thromboembolism (*n* = 2) or were not reported (*n* = 2). Only 1 study specified their anatomical target of the tibial nerve (26).

**Table 3:** Response of phantom and residual limb pain and functional outcomes to cryoablation.

Study	Study Size (*n*)	PLP Severity (Mean Score/10)			RLP Severity (Mean Score/10)			Additional outcome(s)
		BL	1–3 m	6 m	BL	1–3 m	6 m	
Fiala et al. (26)	1	9	9	—	9	—	—	Improved quality of life
von Falck et al. (22)	7	—	—	—	8.3	—	—	Patient satisfaction (mean 69%) and willingness to undergo repeat procedure (6/7)
Prologo et al. (23)	21	6.2	2.3	2	—	—	—	Improved Roland Morris Disability questionnaire score (BL: 11.3; 1–3 m: 3.3) and willingness to undergo repeat procedure (18/21)
Ramsook et al. (25)	1	—	—	—	—	—	0	Improved prosthetic use
Moesker et al. (24)	5	8.6	—	4	—	—	—	Improved prosthetic use (5/5)

PLP, phantom limb pain; RLP, residual limb pain; BL, baseline; and m, month.

Across 3 reports and series (*n* = 27 patients) (23, 24, 26) cryoablation improved PLP at 6-months [3.9 (95% CI 3.7–4.1); *p* < 0.0001]. This is in contrast to the single cross over randomized control trial identified in our search (27). This study randomized 144 patients (43 female) with either transfemoral (*n* = 43), transtibial (*n* = 92), or ankle (*n* = 9) amputation to receive both sciatic and femoral nerve ultrasound-guided cryoablation or sham treatment. Both groups had a baseline median PLP score of 5.0 that did not significantly improve at 4-months in either the active treatment [median 4.3 (interquartile range 1.5, 6)] or sham group [median 4.5 (interquartile range 2, 6)].

### Pulsed RFA

3 case series (28–30) and 7 case reports (16, 17, 31–35) evaluated pulsed RFA (Table 4). These studies included patients with amputations due to trauma (*n* = 9), PVD (*n* = 2), neoplasm (*n* = 1), and infection (*n* = 2). No etiology reported for 2 case reports. The anatomical targets for ablation included cervical nerve roots (*n* = 1), brachial plexus (*n* = 1), lumbar dorsal root ganglia (DRG; *n* = 2), femoral (*n* = 3), sciatic (*n* = 5), tibial (*n* = 1), and fibular (*n* = 1).

**Table 4:** Response of phantom and residual limb pain to and functional outcomes pulse radiofrequency ablation.

Study	Study size (*n*)	PLP Severity (Mean Score/10)			RLP Severity (Mean Score/10)			Additional outcome(s)
		BL	1–3 m	6 m	BL	1–3 m	6 m	
Brzeziński et al. (31)	1	4.5	0	0	—	—	—	None
Li et al. (32)	1	8	2.5	—	—	—	—	None
Zheng et al. (16)	1	6	1 m: 0.6	1.8	—	—	—	None
			3 m: 1.8					
Zeng et al. (17)	1	9	1 m: 1.8	1.8	8	1	—	None
			3 m: 1.8					
Kim et al. (33)	1	—	—	—	8.5	—	2.5	Improved prosthetic use
Imani et al. (28)	2	5.5	^—^	^—^	—	—	—	None
Restrepo-Garces et al. (34)	1	—	—	—	8	1 m: 0.8	0.8	Improved prosthetic use
						3 m: 0.8		
West et al. (29)	4	8	—	0	8.3	^—^	2.1	Improved prosthetic use (4/4), decreased frequency of PLP (1/4) and decreased phantom limb sensation (1/4)
Ramanavarapu et al. (30)	2	—	—	—	—	—	—	Improved prosthetic use
Wilkes et al. (35)	1	7	0	—	—	—	—	None

PLP, phantom limb pain; RLP, residual limb pain; BL, baseline; and m, month.

In 4 studies (*n* = 6 patients) (17, 29, 33, 34), pulsed RFA improved RLP at 1-month (7.8 (95% CI 7.1–8.5; *p* < 0.0001) and 6-months [6.4 (95% CI 4.7–8.1; *p* < 0.0001)]. In 7 studies (*n* = 10 patients) (16, 17, 28, 29, 31, 32, 35), pulsed RFA improved PLP at 1-month [5.9 (95% CI 4.0–7.7; *p* < 0.0001)] and 6-months [5.6 (95% CI 3.7–7.5); *p* < 0.0001]].

### Complications

A total of 33 patients underwent thermal RFA, with 16 patients having at least 2 ablation procedures. Across all patients, no adverse events were reported. Among the 156 patients that had cryoablation, 1 patient had a fall as a result of a temporary motor block (27), 1 developed prolonged nociceptive pain (26), 6 required post-procedure analgesia with non-steroidal anti-inflammatories (23), and 1 experienced transient skin erythema (22). In the 15 patients that had pulsed RFA, including two patients with repeat procedures, no adverse events were reported.

## Discussion

This is the first review to comprehensively assess the treatment of both upper and lower extremity post-amputation pain with percutaneous ablation. Overall, we identified 19 case series and reports that all demonstrated improvement in post-amputation pain following ablation. Recognizing the small sample size and heterogeneity of the underlying studies, our pooled analysis showed a signal for improved RLP at both 2 and 6-weeks post- thermal RFA. We also showed improved RLP and PLP at 1 and 6-months post-pulsed RFA. While we did also show improvement in PLP at 6-months following cryoablation across case series and reports, this not supported by the randomized control trial identified in our review. There were no major complications reported following thermal and pulsed RFA across all studies studies. In the randomized control trial assessing cryoablation, only a single patient experienced a fall as the result of a temporary motor block, while none of the case reports or case series reported significant complications. Our risk of bias assessment highlighted the inclusion of high-quality case reports, case-series demonstrating moderate risk, and a single moderate risk randomized control trial. Opportunities for bias across case reports and series were mainly related to inconsistencies in reporting of participant inclusion, suggesting an opportunity for an underlying publications bias. As well, inconsistent reporting of adverse events across case reports and series suggests the need to clearly assess the safety profile of thermal and pulsed RFA in well controlled experimental studies, similar cryoablation. Overall, our results suggest that thermal and pulsed RFA may be safe and effective treatments for post-amputation pain.

The discordance between our pooled analysis for cryoablation and the randomized control trial results by Ilfeld et al. (27) may simply be the result of the significantly lower sample size in our pooled analysis and a true type I error. However, Ilfeld et al. (27) did report a clinically meaningful improvement in PLP in almost a third of their control group. This represents a placebo effect or a sustained response to the pre-ablation diagnostic block, and masked a significant response to cryoablation. This study also specifically targeted the sciatic and femoral nerves instead of peripheral neuromas. Given the diameter of these nerves proximally, it is possible that the entirety of the nerve was not sufficiently cooled below the required −20 degrees Celsius to induce a second-degree Sunderland lesion and Wallerian degeneration (36). Insufficient cooling across the nerve, could instead result in a neuropraxic injury and aggravate post-amputation pain (37, 38).

Thermal RFA utilizes extreme heat (commonly around 80 °C), to induce local protein denaturation and coagulative necrosis (39). Lesion size is usually limited by increased tissue impedance from charring. Employing a clockwise pattern with multiple lesions can allow for ablation of large neuromas and/or disrupt continuity of even large proximal nerves. Alternatively, pulsed RFA uses a substantially lower temperature (most often 42 °C) to disrupt peripheral nerve function. Lower temperature radiofrequency pulses have been shown to modulate intracellular protein activity, ion channel and synaptic receptor function, neurotransmitter release, and local inflammatory cytokines (40). Aberrant activity of peripheral nerves/neuromas and peripheral sensitization have been implicated in both RLP and PLP. Therefor pulsed RFA in particular may offer a pathophysiologic advantage for treating post-amputation pain compared to cryoablation or thermal ablation (5).

Peripheral nerve block has previously been used as a minimally invasive treatment for post-amputation pain. Combined corticosteroid and local anesthetic is hypothesized to stabilize the neural membrane, decrease spontaneous firing, and reduce local neuroinflammatory mediators (41). A one-time injection has demonstrated highly variable results with up to half of patients deemed non-responders (42). Alternatively, a randomized control trial of 144 patients with upper or lower extremity amputations and PLP demonstrated a modest benefit of continuous perineural infusion at 4-weeks post-infusion (2.4 ± 3.0) compared with baseline (median 5 (interquartile range 4, 7) (43). Our pooled analysis showed a larger mean decrease at 4-weeks after pulsed RFA [7.8 (95% CI 7.1–8.5; *p* < 0.0001)], without the added complexity of a prolonged infusion. A recent review assessed the effectiveness of TMR and RPNI in both upper and lower extremity amputees (9). When used as a prophylactic treatment, TMR prevented RLP in 25%–100% and PLP in 45%–87% of patients, whereas one study showed RPNI prevented RLP in 100% of patient and PLP in 49% of patient (9). While these results do suggest that prophylactic treatment should be considered at the time of amputation, up to 31% of patients experienced post-operative complications such as infection, delayed wound healing and follow-up unplanned surgery (9). This may be particularly relevant to patients with PVD related amputations where a minimally invasive percutaneous approach may be preferred.

### Limitations and future avenues of research

There are several limitations of this study. Most importantly, our conclusions are based on heterogeneous studies that are small and have an innately high risk of bias. Only one included study employed a randomized controlled design to assess cryoablation (27), and was discordant with the results of our pooled analysis. This highlights the need for future work to recruit larger cohorts of upper and lower extremity amputees to better characterize the efficacy and safety of thermal and pulsed RFA using a randomized design. As well, only one study (23) in our review used a standardized tool to assess functional improvement following ablation. Simply reporting increased prosthetic usage, as done by most studies, is a crude metric that does not elucidate a patient's underlying reason for prosthetic non-adherence (e.g., pain, fatigue, skin breakdown). It is likely insufficient to characterize the functional impact of treatments such as percutaneous ablation. Future studies should employ standardized patient reported outcome tools, such as the Prosthesis Evaluation Questionnaire, Prosthetic Profile of the Amputee Questionnaire, or Orthotics Prosthetics Users Survey (44). These tools provide a comprehensive assessment of functional status, allowing for a better understanding of the patient reported factors that are affected by percutaneous ablation.

## Conclusions

RLP and PLP is unfortunately extremely common after limb amputation and results in reduced quality of life and functional deficits. Multiple interventions have been trialed including prosthetic adjustment, medication management, corticosteroid injection and surgery, however these either provide insufficient relief or have the potential for significant adverse events. We comprehensively assessed the literature related to percutaneous ablation for treating post-amputation pain. Included studies were small and heterogenous but demonstrated both safety and efficacy of thermal and pulse RFA for the treatment of post-amputation pain. Our pooled analysis also supported the use of cryoablation, but this was not supported by evidence from a prior randomized control trial and thus cannot be recommended for the treatment of post-amputation pain. Future studies should include large randomized control trials to better elucidate the role of thermal and pulsed RFA in the treatment continuum of upper and lower extremity post-amputation pain.
