# Supplementary material for: Treatment of residual and phantom limb pain using percutaneous ablation: a systematic review and meta-analysis
Source: Front Rehabil Sci. 2026 Apr 13;7:1802455. doi: 10.3389/fresc.2026.1802455 (PMC13111287; doi:10.3389/fresc.2026.1802455)
Supplement: Supplementary file 1 [file Table1.docx]

Supplementary Table 1: Risk of bias assessment for case studies

|  | SQ1 | SQ2 | SQ3 | SQ4 | SQ5 | SQ6 | SQ7 | SQ8 |
| --- | --- | --- | --- | --- | --- | --- | --- | --- |
| Fiala et al., 2022 | Y | Y | Y | Y | Y | Y | U | Y |
| Sperry et al., 2021 | N | Y | Y | Y | Y | Y | N | Y |
| Brezeinski et al., 2020 | N | Y | Y | Y | Y | Y | N | Y |
| Li et al., 2018 | Y | Y | Y | N | Y | Y | Y | Y |
| Ramsook et al., 2017 | N | N | Y | Y | Y | Y | Y | Y |
| Zheng et al., 2017 | Y | Y | Y | Y | Y | Y | Y | Y |
| Zeng et al., 2016 | Y | Y | Y | Y | Y | Y | Y | Y |
| Kim et al., 2014 | Y | Y | Y | Y | Y | Y | N | Y |
| Imani et al., 2012 | Y | N | Y | Y | Y | Y | N | Y |
| Restrepo-Garces et al., 2011 | Y | Y | Y | Y | Y | Y | Y | Y |
| Ramanavarapu et al., 2008 | Y | Y | Y | Y | Y | Y | N | Y |
| Wilkes et al., 2008 | Y | Y | Y | U | Y | Y | N | Y |

Y – Yes; N – No; U – Unclear.

Signalling Questions:

SQ1. Were patient’s demographic characteristics clearly described?

SQ2. Was the patient’s history clearly described and presented as a timeline?

SQ3. Was the current clinical condition of the patient on presentation clearly described?

SQ4. Were diagnostic tests or assessment methods and the results clearly described?

SQ5. Was the intervention(s) or treatment procedure(s) clearly described?

SQ6. Was the post-intervention clinical condition clearly described?

SQ7. Were adverse events (harms) or unanticipated events identified and described?

SQ8. Does the case report provide takeaway lessons?

Supplementary Table 2: Risk of bias assessment for case series

|  | SQ1 | SQ2 | SQ3 | SQ4 | SQ5 | SQ6 | SQ7 | SQ8 | SQ9 | SQ10 |
| --- | --- | --- | --- | --- | --- | --- | --- | --- | --- | --- |
| von Falck et al., 2022 | Y | Y | Y | U | U | Y | N | Y | Y | Y |
| Pu et al., 2021 | Y | Y | Y | U | Y | Y | Y | Y | Y | Y |
| Guo et al., 2019 | N | Y | Y | Y | Y | Y | Y | Y | Y | Y |
| Prologo et al., 2017 | Y | Y | Y | U | U | Y | Y | Y | Y | Y |
| Zhang et al., 2017 | N | Y | Y | U | U | Y | Y | Y | Y | N |
| Moesker et al., 2014 | N | Y | N | N | Y | Y | Y | Y | Y | Y |
| West et al., 2010 | N | N | Y | U | U | N | N | Y | Y | Y |

Y – Yes; N – No; U – Unclear

Signalling Questions:
SQ1. Were there clear criteria for inclusion in the case series?
SQ2. Was the condition measured in a standard, reliable way for all participants included in the case series?
SQ3. Were valid methods used for identification of the condition for all participants included in the case series?
SQ4. Did the case series have consecutive inclusion of participants?
SQ5. Did the case series have complete inclusion of participants?
SQ6. Was there clear reporting of the demographics of the participants in the study?
SQ7. Was there clear reporting of clinical information of the participants?
SQ8. Were the outcomes or follow up results of cases clearly reported?
SQ9. Was there clear reporting of the presenting site(s)/clinic(s) demographic information?
SQ10. Was statistical analysis appropriate?

Supplementary Table 3: Risk of bias assessment for randomized control trials

|  | D1 | DS | D2 | D3 | D4 | D5 | Overall |
| --- | --- | --- | --- | --- | --- | --- | --- |
| Ilfeld et al., 2023 | L | L | L | L | L | S | S |

H – High concern; L – Low concern; S – Some concern.

Domains:

D1: Risk of bias arising from the randomization process

DS: Risk of bias arising from period and carryover effects

D2: Risk of bias due to deviations from the intended interventions

D3: Missing outcome data

D4: Risk of bias in measurement of the outcome

D5: Risk of bias in selection of the reported result
